# An Amphiphilic Surface with Improved Thermal Radiation for Water Harvesting

**DOI:** 10.3390/molecules29112672

**Published:** 2024-06-05

**Authors:** Han Wang, Shengtao Li, Ye Zhang, Weihui Wu, Khaled Abdeen Mousa Ali, Changyou Li

**Affiliations:** 1 College of Engineering, South China Agricultural University, Guangzhou 510642, China; wanghan603@scau.edu.cn (H.W.); lishengtao@stu.scau.edu.cn (S.L.); zhangye@scau.edu.cn (Y.Z.); 2 School of Intelligent Engineering, Shaoguan University, Shaoguan 512158, China; 3 College of Agricultural Engineering, Al-Azhar University, Cairo 11751, Egypt

**Keywords:** water harvesting, surface wettability, thermal radiation, amphiphile structure

## Abstract

Water scarcity poses a significant challenge for people living in arid areas. Despite the effectiveness of many bioinspired surfaces in promoting vapor condensation, their water-harvesting efficiency is insufficient. This is often exacerbated by overheating, which decreases the performance in terms of the micro-droplet concentration and movement on surfaces. In this study, we used a spotted amphiphilic surface to enhance the surfaces’ water-harvesting efficiency while maintaining their heat emissivity. Through hydrophilic particle screening and hydrophobic groove modifying, the coalescence and sliding characteristics of droplets on the amphiphilic surfaces were improved. The incorporation of boron nitride (BN) nanoparticles further enhanced the surfaces’ ability to harvest energy from condensation. To evaluate the water-harvesting performance of these amphiphilic surfaces, we utilized a real-time recording water-harvesting platform to identify microscopic weight changes on the surfaces. Our findings indicated that the inclusion of glass particles in hydrophobic grooves, combined with 1.0 wt.% BN nanoparticles, enhanced the water-harvesting efficiency of the amphiphilic surfaces by more than 20%.

## 1. Introduction

Water scarcity is a pressing concern for numerous countries [1,2,3,4,5]. Despite advancements, water harvesting from bioinspired surfaces, such as the back textures of desert beetles, has shown limited efficiency when used as a supplementary water source. This primarily arises from the low harvesting efficiency of droplets on amphiphilic surfaces due to low droplet growth and sliding rates. Improving these rates will require the use of surfaces with controllable textures and continuous access to energy. Furthermore, the properties of droplet harvesting under the interactions between droplet sliding and a sur-face’s thermal properties need to be studied. This would require detecting the vapor condensation details under humid circumstances to understand the water-harvesting dynamics on amphiphilic surfaces. These investigations are crucial for advancing our understanding of water-harvesting technologies to address the challenges posed by water scarcity.

A liquid droplet’s wettability on solid surfaces is its response to multiple forces, such as surface tension, Laplace pressure, and gravity. The surface polarity and free energy of solids are vital indicators influencing a droplet’s nucleation efficiency during water harvesting [6,7,8,9,10]. Wang et al. [11] found that the wettability of different surfaces was due to droplets sliding with different rhythms. Pogorzelski et al. [12,13] suggested that clean metal surfaces have a higher surface tension and hydrophilicity, which can improve the heat exchange efficiency between water vapor and solid surfaces. Song et al. [14] explored the wettability transformation of 304 steel with different surface roughness values under different conditions (temperature, pressure, etc.). They showed that roughness dominated the surface wettability in low-temperature environments (T < 120 °C). However, Zhang et al. [15] proposed that rough heterogeneous surfaces caused contact angle hysteresis. To modify the surface wettability of water-harvesting solids, Zhu et al. [16] proposed a pattern of micro-square holes on a copper surface to amplify the Cassie–Baxter effect and transform the surface from hydrophilic to hydrophobic. Khan et al. [17,18] demonstrated that flash laser scanning changed the wettability of a surface without a chemical process. Romário et al. [19] produced a superhydrophobic material composed of vertically aligned carbon nanotubes, which also improved the water-harvesting efficiency. Yan et al. [20] used dichlorodimethylsilane to modify the surface wettability of SiO_2_ nanoparticles.

To explore the influence of diverse surface wettability properties on water-harvesting efficiency, Deng et al. [21,22] determined the relationship between the macroscopic thermal properties of solids and the phase transition efficiency of vapor condensation by analyzing the nucleation, growth, merging, and removal of condensed droplets on surfaces with different wettability values. Liu et al. [23] found that the hydrophilicity or hydrophobicity of inclined surfaces promoted droplet sliding during water harvesting. By constructing nanostructures on the surface of an aluminum metal mesh to control the surface wettability, Kang et al. [10] proved that a hydrophilic surface significantly promoted the droplet sliding efficiency. Especially for surfaces with an active cooling function, a hydrophobic surface promoted their water-harvesting efficiency in a high-humidity environment.

Many studies have prepared bionic surface structures inspired by special surface structures, such as the back of desert beetles [24,25,26,27], the surface of lotus leaves [28], and the surface of pitcher plants [29]. These approaches have concentrated condensed droplets on surfaces [30]. The inflation and migration of droplets can be modified to improve the water-harvesting efficiency, which is expected to become an important supplemental water source [31]. Benda et al. [32] prepared an amphiphilic surface called PMMDBAB-PDMS using UV curing. The PDMS content in the pre-polymers affected the contact angle of the solid surfaces. Yan [33] fabricated a prototype fog collector to improve the droplet shedding frequency by capturing mini droplets spontaneously and rapidly passing them through the pores. Because the chemical properties and morphology of a solid surface greatly affect the condensation efficiency of the droplets, Huang et al. [34] simulated the condensation process of droplets on an amphibious surface by using the Lattice–Boltzmann method. They proposed a micro-nanostructure with a higher condensation efficiency. Sun et al. [35] proposed a coating and immersion method to prepare a hydrophilic/superhydrophobic bionic water-harvesting surface and then proved that adjusting the size and distance of the hydrophilic spots promoted the water-harvesting efficiency. Inspired by the gas trapping of underwater spiders, Zhang et al. [36] proposed an amphiphilic biological hydrogel with adjustable wettability. They demonstrated that the amphiphilic mesh had more water-harvesting advantages than a traditional mesh. Inspired by the water-collecting structure of cactus spines, Zhang et al. [37] proposed an amphiphilic surface with an asymmetrical structure. They found that the smooth water-collecting channels promoted the sliding of coalesced droplets. However, water harvesting is the result of continuous energy exchange among water vapor, a solid medium, and the environment. Simply optimizing the surface texture to facilitate droplet movement has a limited impact on the water-harvesting efficiency overall.

Zhang et al. [38] designed a 3D sandwich structure composed of a hydrophilic hydroxy-rich cellulose layer, a water-conducting layer, and a hydrophobic layer to obtain a water-harvesting structure with mycelium filaments that collected water from the air. Nguyen et al. [39] synthesized a super-amphiphilic microgroove surface with adjustable wettability using fused deposition modeling (FDM). The super-amphiphilic surface improved the mobility of water droplets. Overall, although amphibious surfaces are beneficial for water harvesting, the water-harvesting efficiency, related to the condensation and movement of droplets, which are influenced by the amphibious surface texture and wettability, requires further study.

This paper presents a novel approach to enhancing the water-harvesting efficiency through the development of a new process to construct a spotted amphiphilic surface with superior thermal radiation properties. By accelerating the droplet sliding and augmenting the surface emission power, the water-harvesting ability of an amphiphilic surface was significantly improved. A new experimental platform, equipped with a self-designed C++ program for automated recognition of minute weight fluctuations caused by droplet movements, was established to investigate the imperceptible process of vapor condensation and its correlation with the solid surface’s characteristics. Our findings indicate that improving the surface energy emission of the hydrophobic region, particularly through the addition of nanoparticles, play a crucial role in enhancing the water-harvesting efficiency in a natural environment. However, changes to the thermal radiation properties of the hydrophobic regions may lead to a surface wettability fluctuation. Specifically, the incorporation of boron nitride (BN) nanoparticles (1.0 wt.%) with Polydimethylsiloxane (PDMS) increased the droplet mobility on the hydrophilic spots and enhanced the emission power of the surfaces, thereby promoting water harvesting on spotted amphiphilic surfaces.

## 2. Theoretical Basis

### 2.1. Design Rationale 1: Thermal Radiation and Emission Features on Amphiphilic Surfaces

Although amphiphilic surfaces accelerate the vapor concentration and sliding processes according to texture optimization, it is difficult to develop an amphiphilic surface that maintains a constantly high water-harvesting efficiency because the surface temperature dramatically increases after receiving energy from condensed vapor. Thus, amphiphilic surfaces must deliver energy by thermal radiation. Therefore, the thermal output efficiency of the surfaces was vital for their water harvesting, which was determined by the emissivity of the amphiphilic surfaces according to the Stefan–Boltzmann law [40]:

$$E_{amphiphilic}=\varepsilon_{amphiphilc}\sigma T^{4}$$

where $\varepsilon_{amphiphilc}$ is the emissivity of an amphiphilic surface.

**Figure 1:**
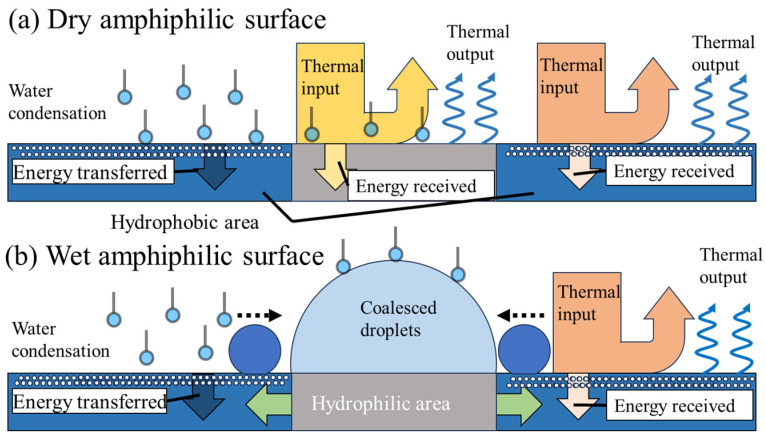
Energy interaction between moisture.

Figure 1 shows that the surfaces received different amounts of energy from condensation and atmospheric radiation under dry and wet conditions. Figure 1a displays that the thermal radiation input from the hydrophilic region (gray areas) and the hydrophobic region (blue area) was dominated by the thermal properties and textures of the dry surfaces. Then, the hydrophilic and hydrophobic parts, displaying high reflectivity, prevented energy from entering via the ambient atmosphere. The emissivity of the amphiphilic surfaces followed the equation $\varepsilon_{amphiphilie}=\varepsilon_{hydrophilie}f_{hydrophilie}+\varepsilon_{hydrophobe}f_{hydrophobe}$, where $\varepsilon_{hydrophilie}$ and $\varepsilon_{hydrophobe}$ are the emissivity of the hydrophilic and hydrophobic surfaces, and $f_{hydrophilie}$ and $f_{hydrophobe}$ are the area fractions of different substances ($f_{hydrophilie}$ + $f_{hydrophobe}$ = 1) on the surface, respectively.

Figure 1b indicates that thermal radiation’s absorption by the hydrophilic region (gray areas) was prevented by concentrated droplets, and the emissivity of the amphiphilic surfaces was mainly determined by the hydrophobic region on the wet regions according to the equation $\varepsilon_{amphiphilie}=\varepsilon_{hydrophobe}f_{hydrophobe}$. Meanwhile, the energy received from condensed vapor on the hydrophilic area was transferred to the hydrophobic area because the emissivity of the hydrophilic area was diminished by the droplets on its surface. 

### 2.2. Design Rationale 1: Surface Mechanicss

**Figure 2:**
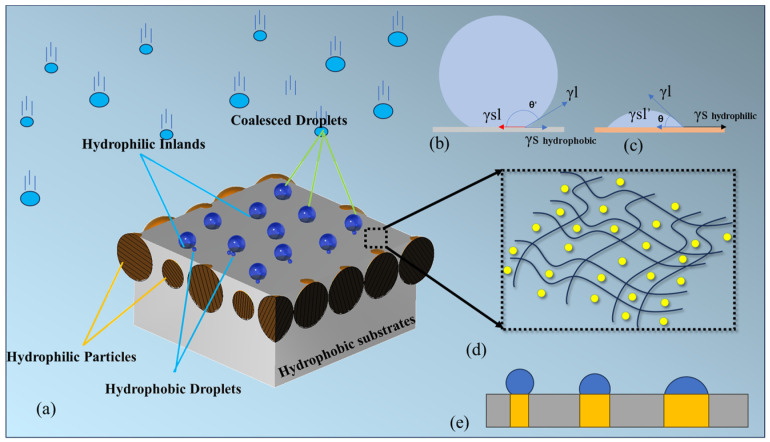
(**a**) Diagram of coalesced droplets on amphiphilic surfaces. (**b**) Diagram of droplets adhered to hydrophobic surface. (**c**) Diagram of droplets adhered to hydrophilic surface. (**d**) Hydrophobic groove mixed with nanoparticles. (**e**) Diagram of droplets’ status on different hydrophilic islands.

The island-like amphiphilic surfaces in Figure 2a require careful design of both their surface wettability and droplet mobility to obtain a higher water-harvesting efficiency. When the surface tension of solids is less than the attracted force among the water molecules, the coalesced droplets tend to form a spherical shape, as shown in Figure 2b. This surface is considered non-wettable. When the surface tension of the solids is larger than the molecular forces, the droplets on the solid surface will show a tendency to expand outwards, as shown in Figure 2c. This surface is considered wettable. Here, we refer to a surface as “hydrophobic” when θ > 90° or “hydrophilic” when θ < 90° [41].

The contact angle of droplets on a solid surface was used to evaluate the surface wettability and droplet mobility [42]. Figure 2e illustrates the relationship between the surface texture and droplet mobility. The droplets coalesced on a smaller hydrophilic island can slide off earlier, as the surrounding hydrophobic grooves prevent the droplets from spreading [43]. Figure 2d shows a hydrophobic area with mixed nanoparticles to improve its energy emission abilities [44,45].

## 3. Results and Discussion

### 3.1. SEM Results

**Figure 3:**
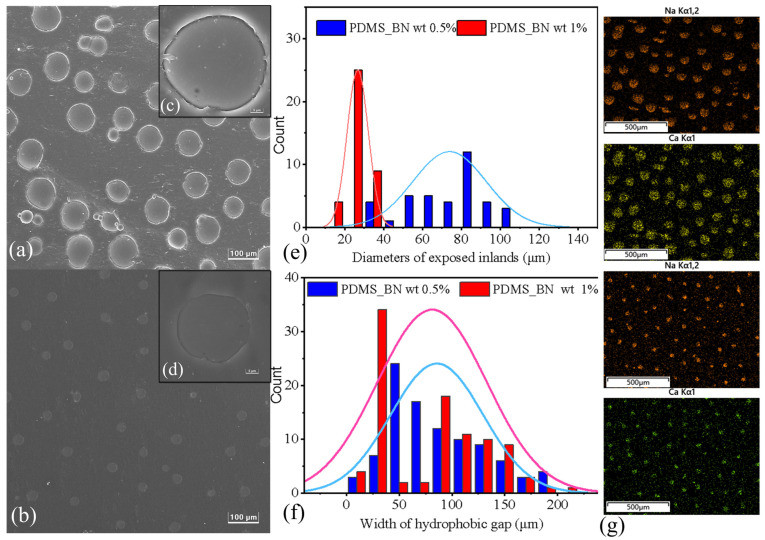
(**a**) SEM image of an amphiphilic surface with an admixture of 0.5 wt.% BN, (**b**) SEM image of an amphiphilic surface with an admixture of 1.0 wt.% BN, (**c**) SEM image of hydrophilic islands on an admixture of 0.5 wt.% BN on an amphiphilic surface, (**d**) SEM image of hydrophilic islands on an admixture of 1.0 wt.% BN within amphiphilic surface, (**e**) normal distribution of hydrophilic island diameters, (**f**) normal distribution of hydrophobic grooves, (**g**) EDS results of Na and Ca in amphiphilic surfaces.

SEM was used to observe the morphological characteristics of the prepared amphiphilic surfaces with 0.5 wt.% and 1.0 wt.% of the nano-admixtures to enhance the mobility of the droplets in the hydrophobic grooves. The results in Figure 3a,b show that islands were closely surrounded by PDMS solids to form amphiphilic surfaces with different textures. The EDS results displayed the Ca and Na elements distributed on the amphiphilic surfaces to indicate the locations of the emerged glass islands. However, the diameters of the hydrophilic islands on the surface mixed with 0.5 wt.% of the BN nano-admixture (Figure 3c) were significantly larger than those of the BN 1.0 wt.% mixture (Figure 3d). This will have affected the water-harvesting efficiency. We measured the parameters of the hydrophilic islands and the hydrophobic grooves on the different surfaces using the Nano Measurer program and obtained their normal distributions.

The statistical results (Figure 3e,f) showed that the hydrophilic island size was much smaller than the hydrophilic particle diameter (100 μm) in both cases. Therefore, the hydrophilic particles could become tightly stuck in the cured PDMS. The size distribution of the hydrophilic islands of the 0.5 wt.% BN sample was mainly concentrated in the range of 30–100 μm, while the size distribution of the hydrophilic islands of the 1.0 wt.% BN sample was uniformly distributed in the range of 20–40 μm. The former was significantly smaller than the latter. Although the hydrophobic groove widths of the 0.5 wt.% BN sample and the 1.0 wt.% BN sample were in the same range, the groove widths of the former were closer to a normal distribution, while the latter was mainly concentrated at 30 μm and 50 μm. Therefore, the latter provided more precise control of the width of the inlet hydrophobic grooves. We predicted that the structural features on the amphiphilic surface of the 0.5 wt.% BN sample would negatively impact the droplet sliding and thermal emissivity when wet.

### 3.2. AFM Result

**Figure 4:**
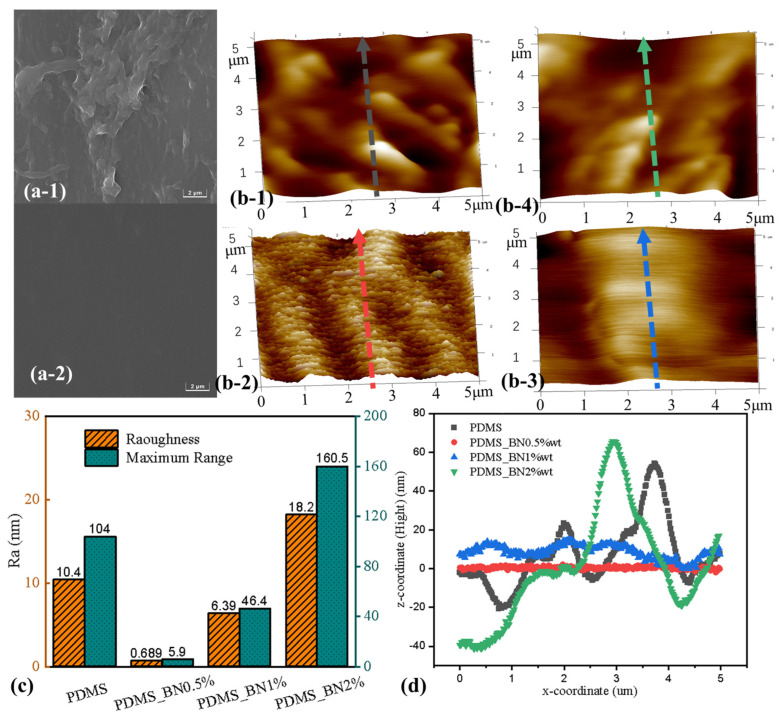
(**a**-**1**) SEM images of hydrophobic grooves on an amphiphilic surface without the nano-admixture and (**a**-**2**) with an admixture of 0.5 wt.% BN. (**b**-**1**) AFM images of hydrophobic grooves on an amphiphilic surface without the nano-admixture and (**b**-**2**) with an admixture of 0.5 wt.% BN. (**b**-**3**) SEM images of hydrophobic grooves on an amphiphilic surface with an admixture of 1.0 wt.% BN and (**b**-**4**) with an admixture of 2.0 wt.% BN. (**c**) Statistics of roughness and maximum altitude difference on amphiphilic surfaces. (**d**) The surface fluctuation details of 4 lines on amphiphilic surfaces.

The SEM images of the amphiphilic surfaces showed that the morphological features of the hydrophobic region in the amphiphilic surface were impacted by the cured PVP adhesive on the pre-templated surface (Figure 4a-1). The roughness of the hydrophobic surface was significantly reduced after adding 0.5 wt.% BN (Figure 4a-2). The nanoscale morphological features of the hydrophobic region on the amphiphilic surface were observed using AFM, which showed that the addition of different proportions of BN (0.5 wt.%, 1.0 wt.%, and 2.0 wt.%) to the PDMS pre-polymers produced different structures on the amphiphilic surfaces. The surface structure of the cured pure PDMS pre-polymer (Figure 4b-1) demonstrated a surface roughness of 10.4 nm and a maximum height difference of 104 nm (Figure 4c). The hydrophobic surface of PDMS_BN 0.5 wt.% (Figure 4b-2) was the smoothest, with a surface roughness of only 0.689 nm and the lowest maximum height difference (5.9 nm). In contrast, the 1.0 wt.% BN surface (Figure 4b-3) was moderately rougher (6.39 nm), with a maximum height difference of 46.4 nm. The hydrophobic surface of the PDMS_BN 2.0 wt.% sample (Figure 4b-4) was rougher than all the other surfaces (18.2 nm), with a maximum height difference of 160.5 nm. We compared the characteristics of continuous height variation for different surfaces by extracting the section profiles in the center of the sample height map (Figure 4d) and found that PDMS_BN 2.0 wt.% significantly reinforced the surface topography of the pre-polymerized template. The peak and valley values of its grooves were significantly higher than those of the original rough PDMS sample. This was because the pre-polymers of the PDMS_BN 1.0 wt.% and PDMS_BN 0.5 wt.% samples filled the surface nanogrooves of the pre-polymerized templates during polymerization, which reduced the surface roughness of the PDMS. The PDMS_BN 0.5 wt.% sample had the most obvious smoothing effect for hydrophobic grooves. Mixing PDMS with BN increased the smoothness of the cured PDMS surface and intensified the thermal radiation efficiency of the hydrophobic area, which was more conducive to droplet sliding and cooling of the surface.

### 3.3. Static Contact Angle Test and XRD Results

During the preparation of amphiphilic surfaces, any additives (glass particles and BN nanoparticles) theoretically reduce the degree of polymerization of PDMS, thus affecting the contact angle. However, when glass beads emerge on the surface, their inherent hydrophilicity influences the contact angle test results. To test the effect of hydrophilic particles and BN nanoparticles on the hydrophobic regions (PDMS), an equal amount of hydrophilic glass beads was mixed into the PDMS. Subsequently, XRD tests and contact angle tests were performed on the surface. The results showed that although XRD only detected the influence of BN on the characteristic peaks of PDMS at the PDMS surface, the internally mixed glass beads also affected the wettability of the hydrophobic regions.

**Figure 5:**
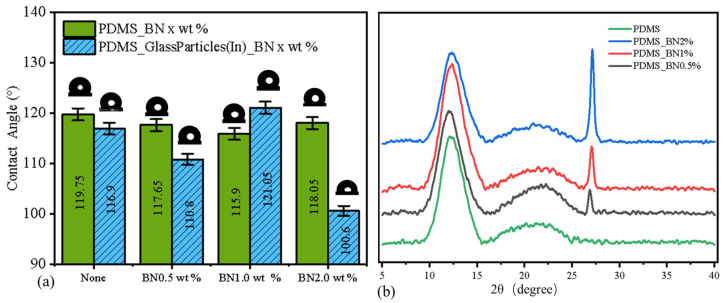
(**a**) The static contact angle results for hydrophobic grooves. (**b**) XRD results of hydrophobic grooves.

The contact angle test was used to reveal the surface wettability and mobility of droplets. We randomly chose three points on each sample to test the wettability of the smooth PDMS surface and the surfaces with different ratios of nanoparticles. This was performed to evaluate the influence on the surface wettability caused by inserting the nanoparticles and glass particles. We characterized the static CA of these amphiphilic surfaces with different volumes of BN nanoparticles using an automated goniometer at ambient conditions with a 5 mL water droplet. The results in Figure 5a show that the green columns represent the results for the PDMS composite surfaces prepared by mixing PDMS with BN admixtures (different ratios), and the blue columns represent the PDMS composite surfaces prepared by mixing PDMS with BN admixtures (different ratios) and glass particles (same mass). The wettability test results indicated that nearly all contact angle of the samples with additives were lower than those of the pure PDMS surface. However, for the samples without added glass particles, the contact angle tended to decrease and then increase as the percentage of BN increased from 0.5% to 2%. The contact angle of the sample with 2.0% BN was very close to the hydrophobicity of the original PDMS (0.4°). The contact angle trend for the samples with added glass particles (blue column) was it being significantly higher than that of the original sample (green column). The wettability of the PDMS mixed surface was already 1.3° higher than that of the original PDMS at a BN content of 1.0 wt.%. However, a significant decrease in the contact angle (100.6°) was observed when the BN content was 2.0 wt.%.

To further investigate the influence of additives on the PDMS surface, we investigated the crystalline components of the PDMS_BN composite surface using XRD, and the results are shown in Figure 5b. The sharp peak on the right side represents crystalline BN, and the height of the crystalline peak increased significantly upon increasing the addition ratio. The broad peak on the left side is the amorphous peak of PDMS. The amorphous peak of PDMS_BN 0.5% was significantly weaker than that of the pure PDMS samples, while the amorphous peak of PDMS_BN 1% was the strongest among all four samples. The amorphous peak of PDMS_BN 2% had the lowest height among all four samples. The heights of the crystalline peaks increased and then decreased upon increasing the BN addition. This was consistent with the trend observed from the contact angle tests.

The results showed that the additives interfered with the crosslinking reaction of the pre-polymer and increased the hydrophobicity and thermal emissivity of the amphiphilic surfaces. The inserted hydrophilic particles also affected the surface tension of the PDMS area. The hydrophobicity of the PDMS on the PDMS_Glass_BN 1.0 wt.% sample was higher than that of pure PDMS, which increased the mobility of the droplets on the amphiphilic surfaces. However, adding excess nanoparticles may expose BN, which may degrade the hydrophobicity of the PDMS area.

### 3.4. Droplet Coalescence Test Results

**Figure 6:**
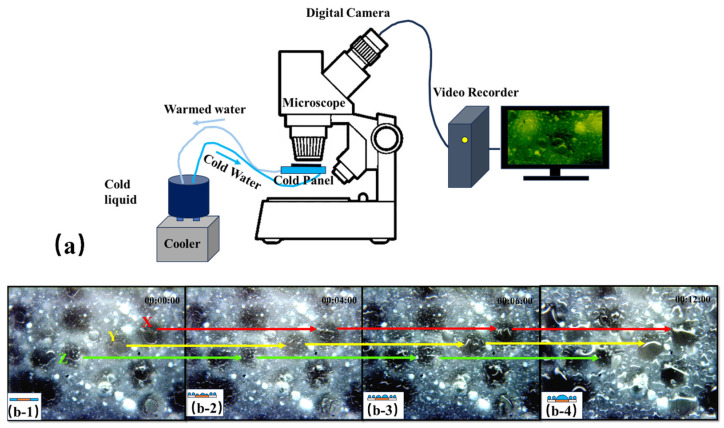
(**a**) Diagram of droplet coalescence test platform. (**b-1**–**b-4**) Droplet inflation and coalescence processes of the amphiphilic surface.

The experimental samples were placed on a copper plate with a lower temperature to observe the vapor concentrating and droplet coalescence processes on the amphiphilic surfaces. Figure 6a shows that the cold panel is equipped with metal tubes to allow cold water to flow, maintaining plate cooling and thereby accelerating the water vapor condensation process on the amphiphilic surface. The water vapor condensation process was observed and recorded using an optical microscope, which provided important evidence for evaluating the water vapor concentrating and merging properties on the amphiphilic surfaces.

The vapor concentration efficiency and droplet sliding behavior were observed over a period of 1 h. Initially, before the vapor concentration (Figure 6b-1), there was a distinct boundary between the hydrophilic and hydrophobic regions of the amphiphilic surface. Subsequently, as water vapor continuously landed on the surface, no significant difference in the droplet sizes between the hydrophilic islands and hydrophobic grooves was observed at around 4 min into the experiment (Figure 6b-2). However, at approximately 6 min (Figure 6b-3), it became evident that droplet coalescence on the hydrophilic islands occurred more frequently than in the hydrophobic region, resulting in larger droplets in the former. By the 12th minute of the experiment (Figure 6b-4), the droplets on all the hydrophilic islands had merged into large droplets, with overflow observed on larger islands (X and Y) and successful coalescence on smaller islands (Z). At this stage, the boundary between the water islands and the hydrophobic gullies became indistinct, and the main driving force for droplet movement in this phase was attributed to the hydrophobic region.

### 3.5. Water-Harvesting Test Results

**Figure 7:**
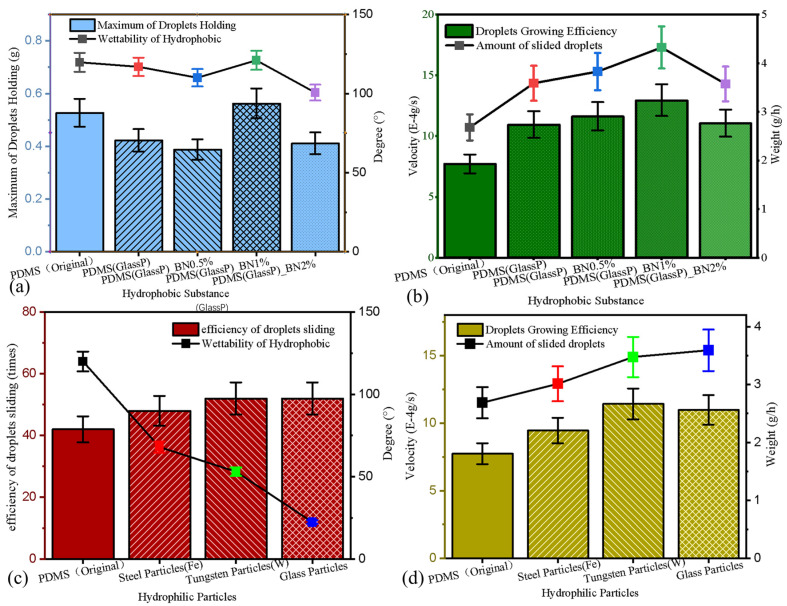
(**a**) The results for surface wettability and droplet dragging ability on amphiphilic surfaces with different volumes of BN nanoparticles. (**b**) The result of droplets sliding and growing on amphiphilic surfaces with different volumes of BN nanoparticles. (**c**) The result of surface wettability and droplet sliding ability on amphiphilic surfaces with different hydrophilic particles inserted. (**d**) The result of droplets sliding and growing on amphiphilic surfaces with different hydrophilic particles inserted.

Figure 7a,c show the maximum weight of the droplets on the samples (bar graph) before sliding events. The line graph represents the static water contact angles of different PDMS surfaces and hydrophilic particulates previously reported [13,44,45]. Figure 7a shows that bigger droplets holding onto the samples was significantly positively correlated with the wettability of the sample surface. Meanwhile, the droplet mass carried by the surface of sample PDMS(GlassP)_BNwt 1.0% was the largest (0.56293 g), which also matched the lowest (121°) wettability of the hydrophobic samples. Conversely, the PDMS(GlassP)_BNwt 0.5% surface carried the smallest droplet (0.38769 g), and the contact angle of PDMS(GlassP)_BNwt 2% was the smallest of all the samples (100.6°). Figure 7c reveals that the higher the frequency of the droplet sliding, the better the wettability of the hydrophilic islands on the amphiphilic surfaces. The highest droplet slippage efficiency (52 times/h) was found on the sample whose hydrophilic island material was glass, and this sample also displayed the most hydrophilic islands. Apart from hydrophobic surfaces (42 times/h), the lowest droplet fall-off rating was observed for the amphiphilic surface with iron particles (48 times/h), which also was the hydrophilic island with the lowest wettability.

Figure 7b,d show the mean vapor concentration speed (bar graph) for each droplet-refreshing cycle on different surfaces. The line graph represents the mass of water vapor collected by different surfaces during the test. Figure 7b shows that the vapor concentration efficiency on the sample surfaces was vital for determining the water-harvesting efficiency of the amphiphilic surfaces. Moreover, the water-harvesting quantity and vapor condensation rate on the amphiphilic surfaces were overall higher than those of the purely hydrophobic samples (2.68 g and 7.73 × 10^−4^ g/s). The surface with the highest water vapor condensation efficiency was sample PDMS(GlassP)_BNwt 1.0% (12.99 × 10^−4^ g/s), which also collected the most water (4.2262 g) during the test (1 h). Except for the hydrophobic samples, PDMS(GlassP)_BNwt 2% collected the smallest amount of water (3.5764 g), and its water-harvesting rate was the same as that of the sample PDMS(GlassP) without the BN admixture (10.97 × 10^−4^ g/s and 11.08 × 10^−4^ g/s). Figure 7d shows a strong relationship between the condensation vapor concentration and water harvesting on the amphiphilic surfaces. The water-harvesting mass and vapor landing efficiency of the amphiphilic surfaces with different hydrophilic island compositions were equally higher than those of the purely hydrophobic samples (2.68 g and 7.73 × 10^−4^ g/s). When the hydrophilic/homophobic ratio of the amphiphilic surface was constant, increasing the wettability dissimilation of the hydrophilic island and hydrophobic grooves improved the water-harvesting efficiency. Although the composite surface with added tungsten particles showed the highest water-harvesting efficiency (11.417 × 10^−4^ g/s), the amphiphilic surface with the highest amount of water harvesting was the one containing added glass particles (3.5915 g). Apart from the hydrophobic samples, the amphiphilic surface with Fe particles harvested the smallest amount of water (3.01 g), and it also concentrated vapor at a lower efficiency than the other two amphiphilic surfaces.

## 4. Materials and Methods

Given these physical insights, we investigated a wide range of nanoparticles and hydrophilic islands. To design an amphiphilic surface with emerged islands, three engineering criteria must be satisfied. First, the candidate particles must be wettable but not soluble in water. Second, the hydrophilic particles must be closely connected to an adhesive until the end of the polymer curing process to produce a surface with emerged islands. Third, the nano-admixture should be inserted and evenly dispersed in the pre-polymer liquid and must also maintain its structural stability and high thermal reflectivity and emissivity. The design principle of such a surface includes the careful selection of a base admixture and particles, as well as structural design. We investigated several particles with different surface tension values, including glass [46], iron [13], tungsten [47], and a different volume of admixtures (BN nanoparticles). These particles can be closely bundled by selecting an appropriate assembly process. All the candidate particles were divided into different groups based on their different diameters.

### 4.1. Design Rationale 3: Amphiphilc Surface Assmbly Method

**Figure 8:**
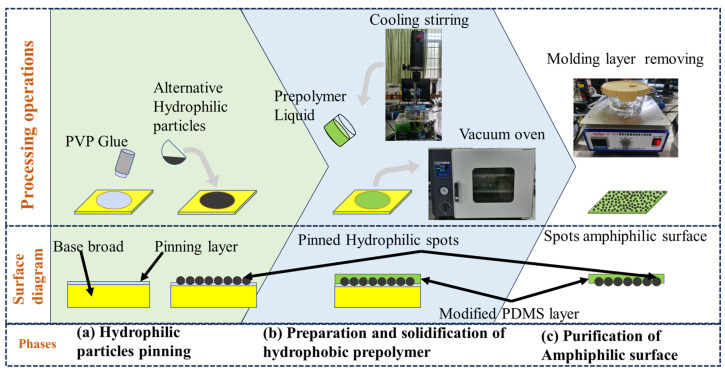
The process flow of amphiphilic surface assembling.

An island-type amphiphilic water-harvesting surface was prepared using a templating method proposed to synthesize a water-harvesting surface that imitated a beetle’s back. The assembly process involved three steps (Figure 8):

(1) Hydrophilic particle pinning: First, PVP semi-solid glue was uniformly applied on the surface of a glass plate. Then, size-screened hydrophilic particles were sprinkled onto the glass glazing plate, which was put into an oven at 60 °C for 30 min to obtain prefabricated templates with fixed hydrophilic islands. The diameter range of the hydrophilic particles was limited to 100–125 μm to eliminate the influence of the amphiphilic surface’s morphology due to the different diameters of the hydrophilic particles.
(2) The preparation and solidification of the hydrophobic prepolymer: The PDMS pre-polymer, crosslinker, diluent, and nanoparticles were mixed at different ratios and mechanically stirred for 10 min at a low temperature until they formed a homogeneous dispersion, which was poured onto the prefabricated templates. The samples were placed into a vacuum-drying oven to perform a two-step solidification process. Firstly, a vacuum was drawn for 30 min at room temperature. After all of the air bubbles were removed, the pre-polymer was cured at 85 °C for 30 min. Finally, solid PDMS composites with an internal amphiphilic structure were obtained.
(3) The purification of the amphiphilic surface: The glass plate applied with solid PDMS was immersed in alcoholic liquid for two hours until the PVP colloid melted and separated from the glass plate. Then, the fallen PDMS solid was placed in an ultrasonic cleaner to clean off the PVP colloid adhered to the PDMS surface to obtain an island-type amphiphilic surface.

### 4.2. Water-Harvesting Performance Test

**Figure 9:**
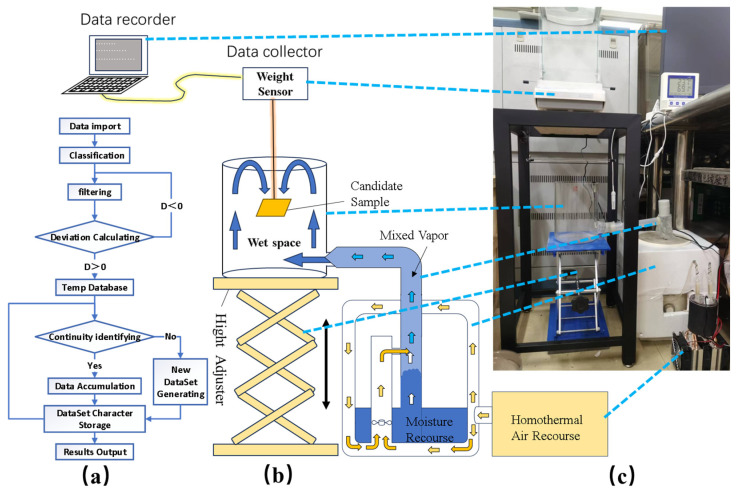
(**a**) Schedule of C++ program design logic; (**b**) diagram of water-harvesting platform; (**c**) set plan of water-harvesting platform.

Amphiphilic surface samples 50 mm × 50 mm in size were suspended in the water collection test rig at an inclination angle of 30° to investigate the influence of the wettability of the hydrophobic grooves and the wettability of the hydrophilic islands for water harvesting. The results on the vapor concentration efficiency and droplet sliding behavior were tested for 1 h. Because the water concentration process is slow and affected by the environment, we designed a water-harvesting performance test platform (Figure 9c) to determine the vapor concentration and droplet sliding on the amphiphilic surfaces. A temperature control function (Figure 9b) was added based on its original structure to reduce the influence of the environmental variables. The stability of the testing environment was improved by controlling the temperature of the liquid in the nebulizer and the temperature of the inlet air. 

The total mass of the droplets (*G_harvesting_*) collected from the different amphiphilic surface structures during the test was used to evaluate the growth and movement of the droplets on amphiphilic surfaces,$\;G_{Harvesting}=\sum_{i=1}^{n}G_{i}$, where *G_Harvesting_* represents the total weight of the droplets dropped per unit of time, and *G*_i_ represents the mass of the droplets dropped each time. Water harvesting is a discontinuous process from water vapor condensation to growth and then to sliding from the surface. The sample weight continues to grow during vapor’s concentration on a surface unless a droplet falls off, in which case the sample weight will drop sharply. These data are detected using an electronic balance and saved in a computer. The weight of the test sample will periodically increase until the next droplets fall. We designed a C++17 program (Figure 9a) to distinguish the weight chain into subsets regarding the weight increasing or decreasing due to the triggers. Then, we recorded the difference between the first and last data points in the subsets as the mass of the droplets dropped (*G_i_*). Finally, the results of the recorded data were output as a report following the chronological order of the droplets dropping. The vapor concentration efficiency during the droplet dropping intervals and the maximum droplets that could be carried on the surfaces were calculated through data post-processing. This provided experimental support to provide a comparison of the water vapor concentrations and sliding efficiency on the amphiphilic surfaces. 

Detailed data on the amphiphilic surfaces, such as their nanostructure and composition, were obtained using SEM, XRD, and AFM to identify the key factors of the amphiphilic surfaces that affected their water-harvesting performance.

## 5. Conclusions

In this study, we systematically screened raw materials and nanoparticles mixed into PDMS to improve the droplet movements and thermal radiation properties. A comparative analysis of various amphiphilic surfaces was carried out to evaluate the droplet coalescence and sliding process to facilitate their water-harvesting efficiency. Our findings highlight the pivotal role of surfaces’ textures and energy emissive ability in promoting complete water harvesting on amphiphilic surfaces. We also observed that the mobility of droplets in misty environments varied according to the surfaces’ wettability. Our research also underscores the significance of the surface tension of hydrophobic grooves, which we modified by incorporating BN nanoparticles and inserting hydrophilic particles during hydrophobic groove curing. This adjustment allowed us to increase the contact angle of PDMS, thereby enhancing the sliding frequency of droplets. Moreover, our investigation revealed that the quantity of added nanoparticles had a crucial effect on the thermal emission power of the amphiphilic surfaces during water-harvesting tests.

## Data Availability

The data presented in this study are available in article.

## References

[1] Overpeck J.T. (2013). The challenge of hot drought. Nature.

[2] McGrath G.S., Sadler R., Fleming K., Tregoning P., Hinz C., Veneklaas E.J. (2012). Tropical cyclones and the ecohydrology of Australia’s recent continental-scale drought. Geophys. Res. Lett..

[3] Dai A. (2013). Increasing drought under global warming in observations and models. Nat. Clim. Chang..

[4] Trenberth K.E., Dai A., van der Schrier G., Jones P.D., Barichivich J., Briffa K.R., Sheffield J. (2014). Global warming and changes in drought. Nat. Clim. Chang..

[5] Grubert E.A., Stillwell A.S., Webber M.E. (2014). Where does solar-aided seawater desalination make sense? A method for identifying sustainable sites. Desalination.

[6] Song Y.-Y., Liu Y., Jiang H.-B., Li S.-Y., Kaya C., Stegmaier T., Han Z.-W., Ren L.-Q. (2018). Temperature-tunable wettability on a bioinspired structured graphene surface for fog collection and unidirectional transport. Nanoscale.

[7] Hou B., Wu C., Li X., Huang J., Chen M. (2021). Contact line-based model for the Cassie-Wenzel transition of a sessile droplet on the hydrophobic micropillar-structured surfaces. Appl. Surf. Sci..

[8] Qin S., Jin Y., Yin F., Wang Z., Bai G. (2022). Can solid surface energy be a predictor of ice nucleation ability?. Appl. Surf. Sci..

[9] Bao X., Zhang M., Li P., Lu J., Yuan G., Zuo Y. (2021). Investigating the surface wettability and surface free energy of sodium silicate-impregnated poplar wood. Wood Mater. Sci. Eng..

[10] Kang J.H., Lee J.-W., Kim J.Y., Moon J.W., Jang H.S., Jung S.Y. (2021). Effect of Mesh Wettability Modification on Atmospheric and Industrial Fog Harvesting. Front. Phys..

[11] Wang S.-W., Peng L., Chen J.-W., Li L. (2021). A comparative study of the self-propelled jumping capabilities of coalesced droplets on RTV surfaces and superhydrophobic surfaces. Chin. Phys. B.

[12] Wu S.-C., Lin Z.-H., Lo C.-K. (2022). Study of super-hydrophilic nanoscale bilayer assembly surface modification and its application to enhance evaporation. Therm. Sci. Eng. Prog..

[13] Pogorzelski S., Boniewicz-Szmyt K., Grzegorczyk M., Rochowski P. (2022). Wettability of Metal Surfaces Affected by Paint Layer Covering. Materials.

[14] Song J.-W., Fan L.-W. (2023). Understanding the effects of surface roughness on the temperature and pressure relevancy of water contact angles. Colloids Surf. A Physicochem. Eng. Asp..

[15] Zhang X., Qin Y. (2019). Contact angle hysteresis of a water droplet on a hydrophobic fuel cell surface. J. Colloid Interface Sci..

[16] Zhu D., Shi Z., Tan X., Zhang J., Zhang S., Zhang X. (2020). Accelerated wetting transition from hydrophilic to hydrophobic of sputtered Cu films with micro-scale patterns. Appl. Surf. Sci..

[17] Khan S.A., Boltaev G.S., Iqbal M., Kim V., Ganeev R.A., Alnaser A.S. (2021). Ultrafast fiber laser-induced fabrication of superhydrophobic and self-cleaning metal surfaces. Appl. Surf. Sci..

[18] Liu M., Li M.-T., Xu S., Yang H., Sun H.-B. (2020). Bioinspired Superhydrophobic Surfaces via Laser-Structuring. Front. Chem..

[19] Pinheiro R.A., Rosa F.M., Volú R.M., de Vasconcelos G., Trava-Airoldi V.J., Corat E.J. (2020). Vertically aligned carbon nanotubes (VACNT) surfaces coated with polyethylene for enhanced dew harvesting. Diam. Relat. Mater..

[20] Yan Y.-L., Cai Y.-X., Liu X.-C., Ma G.-W., Lv W., Wang M.-X. (2020). Hydrophobic Modification on the Surface of SiO_2_ Nanoparticle: Wettability Control. Langmuir.

[21] Deng Z., Gao S., Wang H., Liu X., Zhang C. (2022). Visualization study on the condensation heat transfer on vertical surfaces with a wettability gradient. Int. J. Heat Mass Transf..

[22] Kim D.E., Oh J.S. (2022). Local phase and thermal behaviors in pool boiling on different wettability surfaces. Exp. Therm. Fluid Sci..

[23] Liu X., Zhao X., Lu L., Li J. (2022). Liquid bridges between particles and the hydrophobic or hydrophilic surfaces of solar photovoltaic glass. Sci. Total Environ..

[24] Parker A.R., Lawrence C.R. (2001). Water capture by a desert beetle. Nature.

[25] Yu Z., Yun F.F., Wang Y., Yao L., Dou S., Liu K., Jiang L., Wang X. (2017). Desert Beetle-Inspired Superwettable Patterned Surfaces for Water Harvesting. Small.

[26] Chen Z., Zhang Z. (2020). Recent progress in beetle-inspired superhydrophilic-superhydrophobic micropatterned water-collection materials. Water Sci. Technol..

[27] Zhu H., Huang Y., Lou X., Xia F. (2019). Beetle-inspired wettable materials: From fabrications to applications. Mater. Today Nano.

[28] Dai X., Sun N., Steven O.N., Birgitt B.S., Wang J., Yang S., Wong T.-S. (2018). Hydrophilic directional slippery rough surfaces for water harvesting. Sci. Adv..

[29] Yue H., Zeng Q., Huang J., Guo Z., Liu W. (2022). Fog collection behavior of bionic surface and large fog collector: A review. Adv. Colloid Interface Sci..

[30] Jarimi H., Powell R., Riffat S. (2020). Review of sustainable methods for atmospheric water harvesting. Int. J. Low-Carbon Technol..

[31] Wang B., Zhou X., Guo Z., Liu W. (2021). Recent advances in atmosphere water harvesting: Design principle, materials, devices, and applications. Nano Today.

[32] Benda J., Narikiyo H., Stafslien S.J., VanderWal L.J., Finlay J.A., Aldred N., Clare A.S., Webster D.C. (2022). Studying the Effect of Pre-Polymer Composition and Incorporation of Surface-Modifying Amphiphilic Additives on the Fouling-Release Performance of Amphiphilic Siloxane-Polyurethane Coatings. ACS Appl. Mater. Interfaces.

[33] Yan D., Chen Y., Liu J., Song J. (2023). Super-Fast Fog Collector Based on Self-Driven Jet of Mini Fog Droplets. Small.

[34] Huang B., Zhang X., Yao Z. (2018). Condensation on solid surfaces with amphiphilic micro-nanostructures by lattice Boltzmann simulation. Chem. Phys..

[35] Sun R., Zhao J., Liu C., Yu N., Mo J., Pan Y., Luo D. (2022). Design and optimization of hybrid superhydrophobic–hydrophilic pattern surfaces for improving fog harvesting efficiency. Prog. Org. Coat..

[36] Zhang M., Zhao T., Yu C., Liu Q., Wang G., Yang H., Yang M., Jiang L., Liu M. (2022). Amphiphilic Pd@micro-organohydrogels with controlled wettability for enhancing gas-liquid-solid triphasic catalytic performance. Nano Res..

[37] Zhang S., Chi M., Mo J., Liu T., Liu Y., Fu Q., Wang J., Luo B., Qin Y., Wang S. (2022). Bioinspired asymmetric amphiphilic surface for triboelectric enhanced efficient water harvesting. Nat. Commun..

[38] Zhang Y., Zhu C., Shi J., Yamanaka S., Morikawa H. (2022). Bioinspired Composite Materials used for Efficient Fog Harvesting with Structures that Consist of Fungi-Mycelia Networks. ACS Sustain. Chem. Eng..

[39] Nguyen V.-T., Park E., Nguyen N.-A., Omelianovych O., Larina L.L., Hussain S.S., Choi H.-S. (2023). 3D-printed plasma-treated super-amphiphilic microgroove surface for outperformance of liquid vertical transportation. Appl. Surf. Sci..

[40] Kozbial A., Trouba C., Liu H., Li L. (2017). Characterization of the Intrinsic Water Wettability of Graphite Using Contact Angle Measurements: Effect of Defects on Static and Dynamic Contact Angles. Langmuir.

[41] Chen Y., Yan D., Liu R., Lu Y., Zhao D., Deng X., Song J. (2022). Green self-propelling swimmer driven by rain droplets. Nano Energy.

[42] Suaste E., Castillo V., González R. (2004). Determination of the phase transition in Pb_0.88_Ln_0.08_Ti_0.98_Mn_0.02_O_3_ (Ln=La, Sm, Eu) piezoceramics based on the Stefan–Boltzmann law. Mater. Charact..

[43] Wang H., Wang D.T., Zhang X.Y., Zhang Z.Z. (2021). Modified PDMS with inserted hydrophilic particles for water harvesting. Compos. Sci. Technol..

[44] Cai Q., Scullion D., Gan W., Falin A., Zhang S., Watanabe K., Taniguchi T., Chen Y., Santos E.J.G., Li L.H. (2019). High thermal conductivity of high-quality monolayer boron nitride and its thermal expansion. Sci. Adv..

[45] Brodu E., Balat-Pichelin M. (2016). Emissivity of Boron Nitride and Metals for the Solar Probe Plus Mission. J. Spacecr. Rocket..

[46] Vukovic T., Røstad J., Farooq U., Torsæter O., van der Net A. (2023). Systematic Study of Wettability Alteration of Glass Surfaces by Dichlorooctamethyltetrasiloxane Silanization—A Guide for Contact Angle Modification. ACS Omega.

[47] He H., Qu N., Zeng Y. (2016). Lotus-leaf-like microstructures on tungsten surface induced by one-step nanosecond laser irradiation. Surf. Coat. Technol..

